# Patchwork: Alignment-Based Retrieval and Concatenation of Phylogenetic Markers from Genomic Data

**DOI:** 10.1093/gbe/evad227

**Published:** 2023-12-12

**Authors:** Felicia Sandberg, Clara G Köhne, Christoph Bleidorn

**Affiliations:** Department for Animal Evolution and Biodiversity, Georg-August-Universität Göttingen, Göttingen 37073, Germany; Cardio-CARE AG, Medizincampus Davos, Davos Wolfgang 7265, Switzerland; Department for Animal Evolution and Biodiversity, Georg-August-Universität Göttingen, Göttingen 37073, Germany; Department for Animal Evolution and Biodiversity, Georg-August-Universität Göttingen, Göttingen 37073, Germany

**Keywords:** genome skimming, low-coverage sequencing, museomics, phylogenomics, short reads, single-copy genes

## Abstract

Low-coverage whole-genome sequencing (also known as “genome skimming”) is becoming an increasingly affordable approach to large-scale phylogenetic analyses. While already routinely used to recover organellar genomes, genome skimming is rather rarely utilized for recovering single-copy nuclear markers. One reason might be that only few tools exist to work with this data type within a phylogenomic context, especially to deal with fragmented genome assemblies. We here present a new software tool called Patchwork for mining phylogenetic markers from highly fragmented short-read assemblies as well as directly from sequence reads. Patchwork is an alignment-based tool that utilizes the sequence aligner DIAMOND and is written in the programming language Julia. Homologous regions are obtained via a sequence similarity search, followed by a “hit stitching” phase, in which adjacent or overlapping regions are merged into a single unit. The novel sliding window algorithm trims away any noncoding regions from the resulting sequence. We demonstrate the utility of Patchwork by recovering near-universal single-copy orthologs within a benchmarking study, and we additionally assess the performance of Patchwork in comparison with other programs. We find that Patchwork allows for accurate retrieval of (putatively) single-copy genes from genome skimming data sets at different sequencing depths with high computational speed, outperforming existing software targeting similar tasks. Patchwork is released under the GNU General Public License version 3. Installation instructions, additional documentation, and the source code itself are all available via GitHub at https://github.com/fethalen/Patchwork.

**Significance**

Even though current sequencing and computational methods allow for the completion of high-quality genomes for all life on earth, the availability of material for sequencing became a major bottleneck in phylogenomic studies, especially since material stored in museum collections—or during barcoding campaigns—is often not suitable for reconstructing high-quality, highly continuous genomes. At the same time, the output of short-read sequencing machines is increasing, and prices for these techniques are dropping. Short-read data are still routinely used to recover organellar genomes, but this so-called genome skimming approach is rather rarely utilized for recovering single-copy nuclear markers. We present a new software tool called Patchwork for mining phylogenetic markers from highly fragmented genome assemblies, as well as directly from short sequence reads. We demonstrate the accuracy of this new approach and show in a benchmarking study that it also outperforms existing software for similar tasks. Patchwork allows to compile preselected gene sets from low-coverage short-read sequencing data sets and is thereby ideally suited when including material from museum collections into phylogenomic studies.

## Introduction

Advancements in high-throughput sequencing techniques have revolutionized the field of phylogenetics and ultimately our understanding of the tree of life (Lemmon and Lemmon 2013). The availability of genomic and transcriptomic data for basically all desired taxa and for a reasonable price has transformed the field to phylogenomics: genome-scale phylogenetic systematic analyses (McCormack et al. 2013). Some challenges remain, however, as many studies still show incongruent results, low branch support, or lacking resolution (Philippe et al. 2017; Steenwyk et al. 2023). Even though complete genomes are becoming available for more and more eukaryotes, the access to high-molecular-weight DNA is the bottleneck in the quest for sequencing genomes of all life on earth (Blom 2021; Dahn et al. 2022). Nowadays and in the past, most large-scale phylogenomic studies were conducted using either transcriptome sequencing or genome subsampling methods such as target enrichment, which focuses on a set of preselected loci (Bleidorn 2017).

Transcriptome sequencing offers a way to sequence only the expressed portion of a genome without prior sequence knowledge (Stark et al. 2019). Unfortunately, this approach requires freshly collected material or specifically stored material, e.g. deeply flash frozen or in RNAlater. Furthermore, smaller specimens may need to be pooled together to attain sufficient amounts of mRNA, and such practice risks mixing up individuals with undetected genetic variation. Unfortunately, a large amount of collected specimens only exist in natural history museum collections, and most of these are ethanol preserved and thus not usable for transcriptomic studies (Call et al. 2021). As taxon sampling is considered one of the most important factors for accurate phylogenetic tree reconstruction (Heath et al. 2008), it would be missing an opportunity to leave the potential of natural history collections untapped. Target enrichment approaches, on the other hand, require prior knowledge of target sequences (e.g. from well-annotated genomes) for the construction of oligonucleotide probes. Moreover, the number of enriched targets is limited by the number of oligonucleotides included in the enrichment kit of choice, and the efficiency of such approaches decreases as the bait-to-target distance increases (Bragg et al. 2016). Another downside is that the data produced are difficult to reuse for other types of genomic or evolutionary studies.

A viable alternative to assemble taxon-rich phylogenomic data sets is low-coverage whole-genome sequencing (LC-WGS; also known as “shallow genome sequencing” or “genome skimming”) using short-read technologies such as Illumina sequencing (Dodsworth 2015). Relying solely on this approach has been shown to be inadequate for the reconstruction of highly contiguous reference-quality genomes (Rhie et al. 2021). However, due to the introduction of newer sequencing platforms (e.g. Illumina's NovaSeq sequencing platform) short-read WGS became relatively cheap and prices are even expected to drop with Ultima Genomics, another highly competitive sequencing platform entering the market (Simmons et al. 2023). Moreover, short-read sequencing library construction also allows that highly fragmented DNA can be used as input (Hu et al. 2021), thereby enabling the use of material from museum collections from all around the world (Raxworthy and Smith 2021). Consequently, LC-WGS can be used to generate data from various sources of targeted organisms to retrieve marker loci on a genome scale. While this so-called “genome skimming” approach has frequently been used to reconstruct organellar genomes or other high-copy fractions of eukaryote genomes (Richter et al. 2015; Jin et al. 2020), it seems currently underutilized to retrieve single-copy nuclear markers (Liu et al. 2021). One reason is that short-read assemblies of eukaryotic genomes tend to be highly discontinuous, and automated annotation of such large, fragmented genomes remains difficult (Salzberg 2019), as they are characterized by the presence of “genes in pieces,” where introns interrupt coding sequences (Rogozin et al. 2005). Depending on the coverage, short-read draft genomes are characterized by low N50s in the range of few (if at all) kilobase pairs (kb; Salzberg et al. 2012), and consequently, exons of a single gene usually end up on several contigs.

The disuse of genome skimming in large-scale phylogenetics could potentially be ascribed to the lack of suitable data analysis methods (Zhang et al. 2019). Existing software tools for working with LC-WGS data in a phylogenomic context, such as aTRAM 2 (Allen et al. 2017, 2018), ALiBaSeq (Knyshov et al. 2021), and GeMoMa (Keilwagen et al. 2016, 2018), are either written in an interpreted language (e.g. Perl or Python) that does not allow the program to scale well with the large biological data sets that are commonplace today (e.g. aTRAM 2, ALiBaSeq) or need well-annotated reference genomes or transcriptomes (e.g. GeMoMa). A recent addition to the portfolio of available tools for such programs is Read2Tree, which directly infers trees from unassembled data (Dylus et al. 2023).

To address the limitations typically associated when working with genome skimming data, we present Patchwork, an alignment-based tool for mining phylogenetic markers directly from WGS data. Patchwork utilizes the sequence aligner DIAMOND (Buchfink et al. 2021) and is written in the programming language Julia (Bezanson et al. 2017) to achieve the best possible speed, thus allowing Patchwork to scale well with today's genome-scale data sets. In addition, our implementation focuses on ease of use, and our program handles each step in the analysis—from start to finish. Using our new approach, we targeted universal single-copy orthologs (USCOs), which are available based on careful analysis of a curated database (OrthoDB, www.orthodb.org). A set of 954 metazoan-specific USCOs has been validated against 364 metazoan genomes and shown to be indeed (i) single-copy and (ii) nearly universally present (Manni et al. 2021).

## Results

**Fig. 1.**
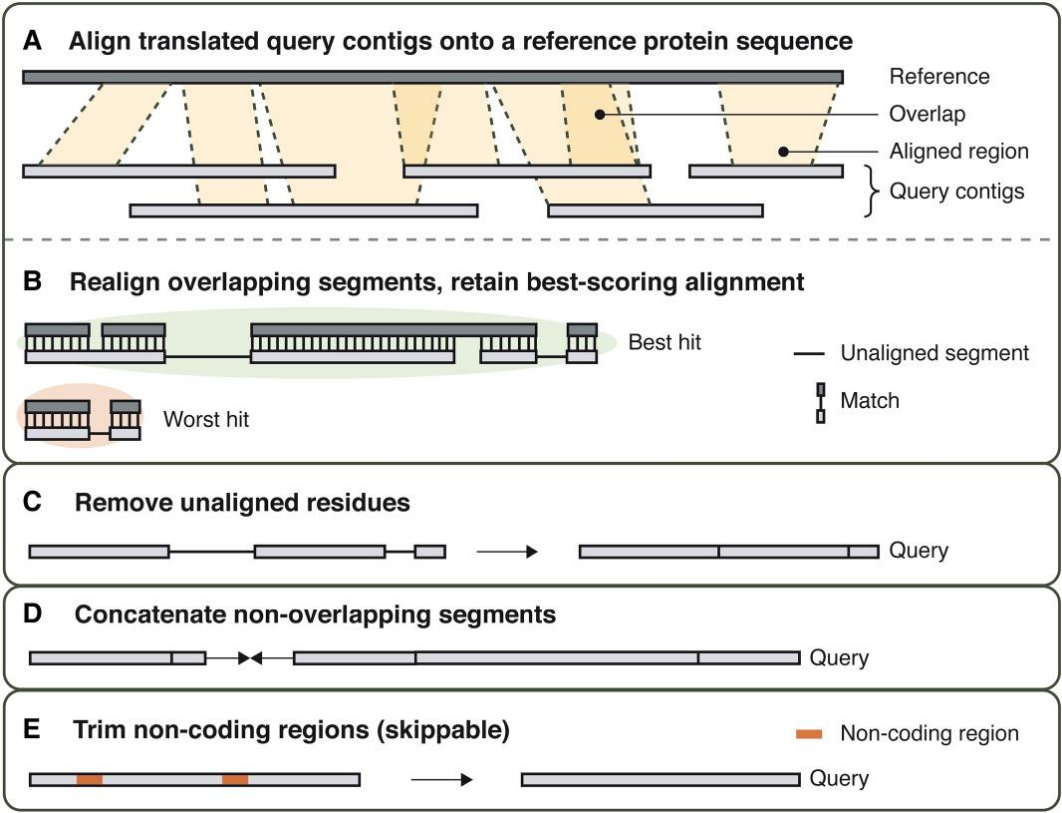
Graphical overview of the Patchwork algorithm. First, a) query sequences are aligned to the provided reference sequence. These alignments may or may not be overlapping. b) Overlapping alignments are realigned but only in the area in which they overlap. The best-scoring alignment is retained while all others are discarded. c) Nonaligned residues are then removed, and d) the remaining regions are concatenated into a single, continuous sequence.

Patchwork is a reference- and alignment-based method for mining phylogenetic markers from WGS data, using either assembled contigs or reads as input (Fig. 1). The aim of Patchwork is to capture multiexon or fragmented genes, scattered across different contigs or reads. One or more reference protein sequences guide the “stitching” process, where the best-scoring translated query nucleotide sequences for any given region are merged into continuous stretches of amino acid sequences. Merged sequences go through a masking step in which unaligned residues, ambiguous amino acid characters (letters that do not determine a unique amino acid; they are B, J, X, or Z, where B = D or N, J = I or L, X = unknown, and Z = E or Q), and stop codons are removed from query sequences. Optionally, the removal of stop codons and ambiguous amino acid characters may be skipped by providing the --retain-stops and --retain-ambiguous flags, respectively. Finally, Patchwork implements a sliding window–based alignment trimming step to remove poorly aligned residues (e.g. due to the presence of putative noncoding regions) from the resulting sequences. The output is available as nucleotide or amino acid sequences.

### Benchmark

To asses performance of our approach, we (i) test an ideal case where the query and reference species are identical, (ii) where the query and reference are 2 distant species, and (iii) compare Patchwork v.0.5.1 with ALiBaSeq v.1.2 (Knyshov et al. 2021) and aTRAM v.2.4.3 (Allen et al. 2017). Throughout these benchmarks, we use Illumina short-read nucleotide sequences from the marine annelid *Dimorphilus gyrociliatus* (accession PRJEB37657 in the European Nucleotide Archive). A highly contiguous (N50 = 2.24 Mb) and complete (95.8% BUSCO genes recovered, metazoa_odb10) annotated version of the compact 73.8-Mb genome of this annelid is publicly available (Martín-Durán et al. 2021).

As we only used short-read data sets at different coverages for our benchmark analyses, we created highly discontinuous assemblies with low N50s as typical for real-world low-coverage genomic data sets. We assembled these sequence reads using SPAdes and subsequently used Patchwork to search for near-USCOs (Seppey et al. 2019), using a preannotated set of USCOs from that same species as a reference. Next, we used that same assembly of *D. gyrociliatus* to search for USCOs, this time using USCOs from the leech *Helobdella robusta* as the reference, a clitellate annelid that diverged at least 400 mya from *D. gyrociliatus* (Erséus et al. 2020). Finally, we compared our program to ALiBaSeq (Knyshov et al. 2021) and aTRAM 2 (Allen et al. 2017). For this comparison, we also subsampled the aforementioned *D. gyrociliatus* short reads in order to simulate various sequencing coverages. We decided not to include the software GeMoMa (Keilwagen et al. 2016) in this comparison, as it heavily relies on the availability of reference genomic or transcriptomic data. Read2Tree (Dylus et al. 2023) has also not been included in the comparisons, as its focus is tree inference and not marker retrieval.

We compared the retrieved translated and stitched contigs, hereafter called “recovered markers,” to the reference *D. gyrociliatus* USCOs. For each reference sequence, the evaluation included percent identical positions out of all aligned positions as well as percent of reference sequence positions covered by the recovered markers. Patchwork automatically generates these statistics and produces a detailed output for each reference as well as an aggregated output over all references.

### Effect of Genome Fragmentation on Accuracy

**Fig. 2.**
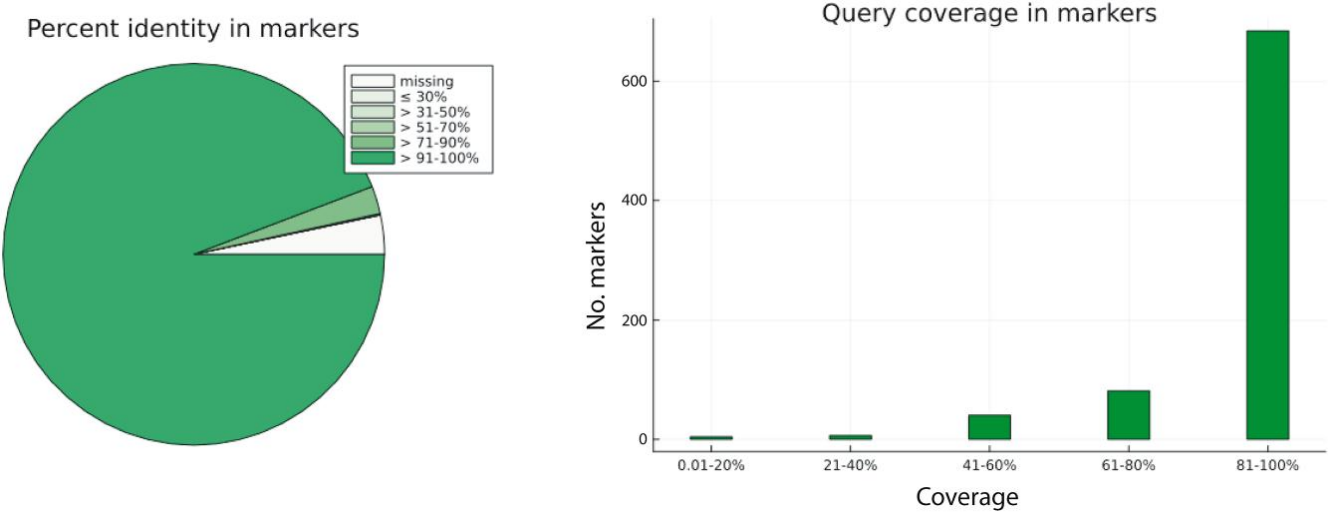
Percent identity and query coverage in markers based on a Patchwork analysis of a SPAdes assembly of *D. gyrociliatus*, targeting 815 single-copy orthologs from the same species.

In the initial setup, we assessed the accuracy of Patchwork using a high-quality query assembly and 815 USCO reference sequences from the same species, *D. gyrociliatus*, and thereby exploring the program's performance for the hypothetical case where the entire set of reference sequences should be recoverable as exactly matching stitched contigs from the query sequences. We retrieved all of the initial 815 markers. On average, 95.9% of all aligned positions were identical matches, with a mean query coverage of 92.2%; this equals a combined measure of 88.4% identical matches for all reference positions, whether aligned to query residues or not (Fig. 2 and Table 1).

**Table 1:** Results from Patchwork when using a *D. gyrociliatus* SPAdes assembly as the query and USCOs from a long-read assembly of *D. gyrociliatus* as a reference

Variable	Mean	Min	Median	Max
Reference length	447.606	77	351.0	2,748
Query length	407.953	27	322.0	2,553
Matches	385.075	24	306.0	2,549
Mismatches	21.207	0	0.0	1,075
Deletions	42.131	0	5.0	1,097
Query coverage	92.181	5.22	98.71	100.0
Identity	95.887	30.91	100.0	100.0

### Effect of Reference Divergence

**Fig. 3.**
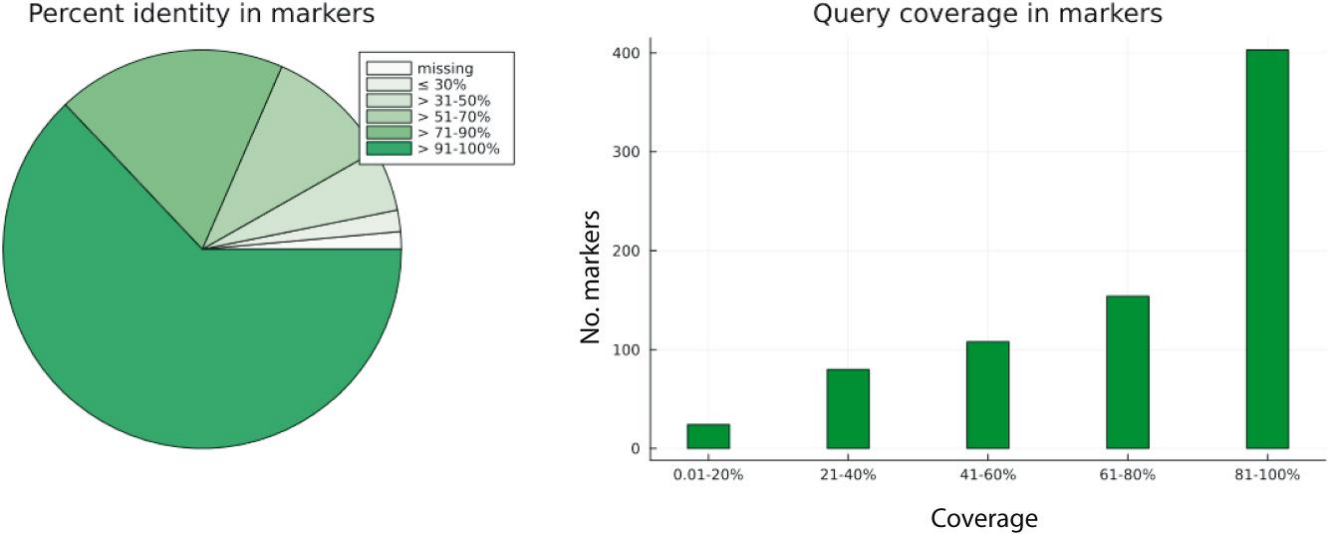
Percent identity and query coverage in markers based on a Patchwork analysis of a SPAdes assembly of *D. gyrociliatus*, targeting 957 single-copy orthologs from the leech *H. robusta*.

In the second iteration, we aligned a set of high-coverage query assemblies against a very distant reference set, in order to estimate the program's performance when using highly divergent sequences as reference. For this purpose, the same *D. gyrociliatus* SPAdes assembly as in the previous evaluation served as query sequence set, and 957 near-USCOs from the annotated genome of the leech *H. robusta* were used as a reference. We retrieved 943 out of the 957 *H. robusta* reference sequences. Of these, 769 successfully aligned back to 1 and only 1 of the 778 *D. gyrociliatus* USCOs that were considered homologous to the *H. robusta* set (Fig. 3 and Table 2). For these 769 retrieved markers, the average percent identity measure was 89%, with a mean query coverage of 74.5%. Put differently, the recovered markers had an average of 67.2% identical matches against all reference positions.

**Table 2:** Results from Patchwork when using a *D. gyrociliatus* SPAdes assembly as the query and USCOs from *H. robusta* as a reference

Variable	Mean	Min	Median	Max
Reference length	448.025	77	351.0	2.748
Query length	309.93	31	259.0	2.326
Matches	249.126	15	195.0	2.326
Mismatches	30.234	0	7.0	355
deletions	8.319	0	2.0	268
Query coverage	74.540	5.41	82.78	100.0
Identity	89.008	25.46	96.99	100.0

The recovered markers were evaluated against the set of 778 *D. gyrociliatus* USCOs that were considered homologous to sequences in the *H. robusta* reference set.

### Program Comparison

**Fig. 4.**
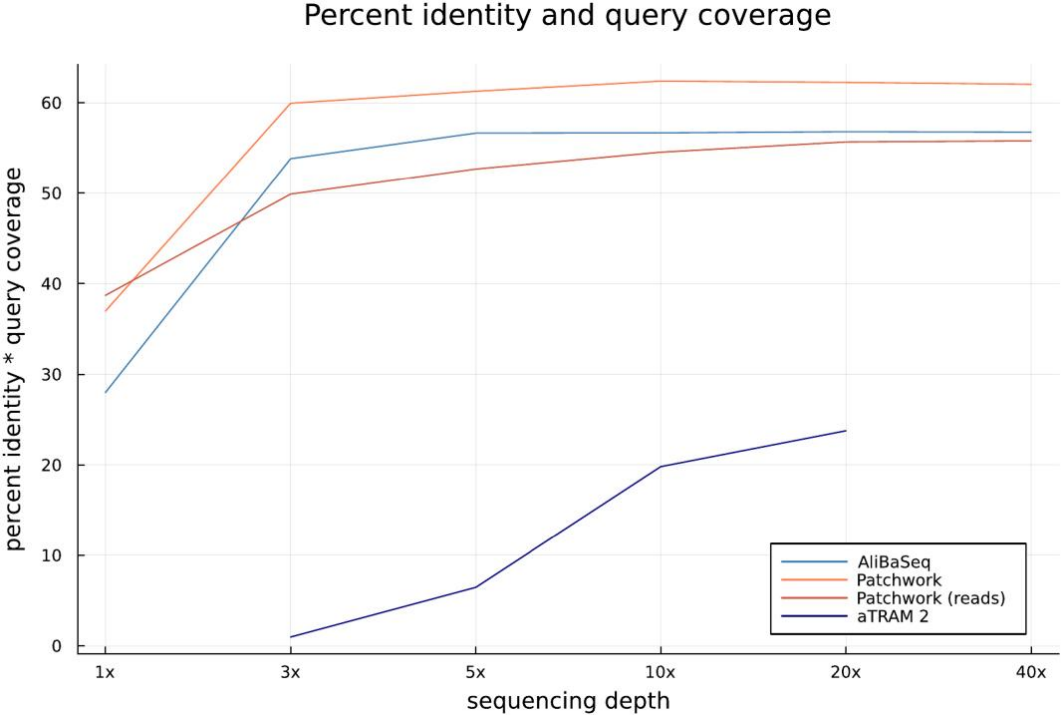
Accuracy and completeness of the recovered marker sequences for the different *D. gyrociliatus* data sets when run against a reference set of *H. robusta* USCOs. Accuracy and completeness were jointly measured as percent identical out of all aligned positions multiplied with the total percentage of aligned nongap positions. This integrated measure avoids a distorted performance estimation, e.g. due to small number of recovered markers but high percent identity in the aligned positions. Patchwork was run with *D. gyrociliatus* assemblies unless indicated differently. ALiBaSeq received assemblies, while aTRAM 2 received reads as input.

In the third setup, we compared the performance and runtime for Patchwork to that of ALiBaSeq (Knyshov et al. 2021) and aTRAM 2 (Allen et al. 2018), using a *D. gyrociliatus* short-read data set at different sequence coverage levels (1×, 3×, 5×, 10×, 20×, and 40×). While Patchwork can use both reads and assembled contigs as an input, ALiBaSeq uses assembled contigs, and aTRAM 2 is read based. Performance was assessed using a combined measure for accuracy and completeness of the recovered USCO markers annotated, hereafter called “total percent identity.” Patchwork with *D. gyrociliatus* assembly data performs best over almost all data sets (Fig. 4), reaching as much as 62% total percent identity for data with at least 10× coverage. Only with a coverage of 1×, *D. gyrociliatus* read-based data seem to be better suited for marker retrieval. Cutoff thresholds during the assembly might lead to discarding part of the sequence data that is retained when using reads, therefore causing the latter to achieve higher query coverage for the 1× data set. Note that query coverage improves especially for read data when tantan masking in DIAMOND is disabled (i.e. by providing --masking 0 as an argument). For data sets with higher coverage, running Patchwork on read data still achieves well over 50% total percent identity. Using read data therefore is a valid option that could be considered if the compute resources necessary for assembling the sequences are scarce. The performance of Patchwork stays approximately constant for data sets with coverages of at least 10×, independent of the used data. By comparison, ALiBaSeq achieves approximately 7% less total percent identity than Patchwork with assemblies for all data sets and performs only slightly better than Patchwork with read data for a coverage over 10×. aTRAM 2, on the other hand, performs comparatively poorly, with a maximum total percent identity of about 22% for the data set with 20× coverage. This is mostly due to the small number of recovered markers; the markers themselves generally have a high percent identity value. For a coverage of 1×, aTRAM 2 was unable to recover any stitched contigs at all. The program was also not evaluated for the data set of 40× coverage as it had not completed within the cluster's maximum runtime of 5 d.

**Fig. 5.**
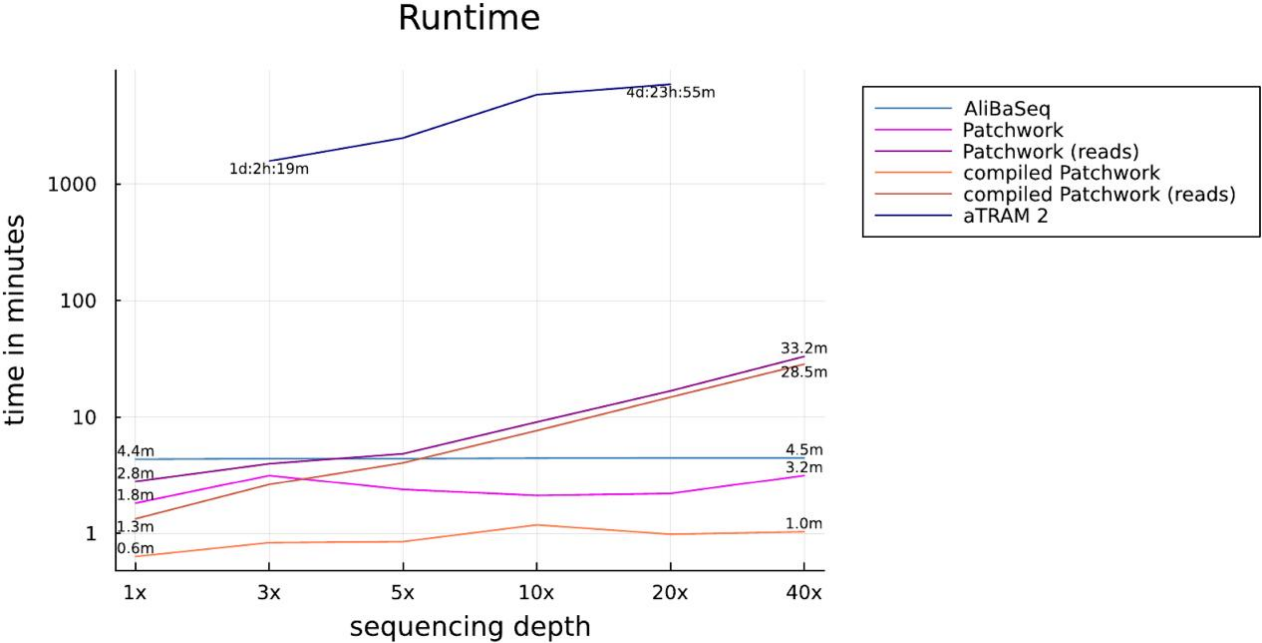
Program runtime for each *D. gyrociliatus* data set. Patchwork was run both as a script and as a compiled program. It received *D. gyrociliatus* assemblies unless indicated differently. ALiBaSeq was run on assemblies, while aTRAM 2 received reads as input.

Both Patchwork and ALiBaSeq are very fast; the programs terminated in under 5 min when using assembly data (Fig. 5). The runtimes fluctuated only slightly between data sets. Using Patchwork with reads required more time for larger data sets, but even for the largest evaluated data set, it finished after half an hour. By comparison, running aTRAM 2 took days for all data sets.

### Patchwork in a Phylogenomic Context

**Fig. 6.**
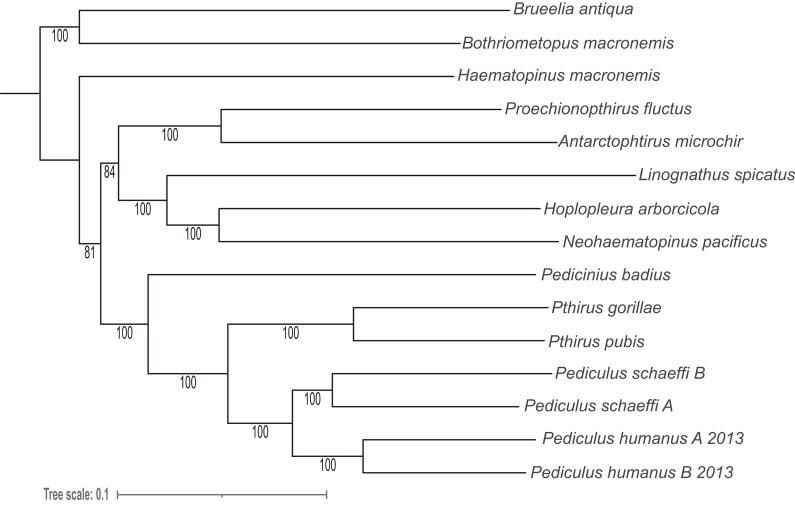
Phylogenetic analysis of Phthiraptera relationships as recovered from a Maximum Likelihood analysis of a combined supermatrix using USCOs as recovered by Patchwork. Analysis was conducted using IQ-TREE 2 including model and partition finding. Bootstrap values from 1,000 pseudoreplicates are given at the branches.

To demonstrate how our software could be utilized in a phylogenomic pipeline, we used it to retrieve a set of 957 metazoan-specific USCOs from a phthirapteran data set (Allen et al. 2017). When reusing a set of 15 lice Operaional Taxonomic Units (Hexapoda and Phthiraptera), we were able to retrieve all 957 USCOs, for all taxa. The resulting alignments contained few gaps for any marker; i.e. most markers were well above the 90% aligned position trimming threshold. The trimmed alignment contained 3,454,320 positions in total, compared to 5,383,303 before trimming (i.e. ∼64% positions were retained after trimming). Our phylogenetic reconstruction resulted in a well-supported tree (Fig. 6), which is largely congruent with the original analysis (Allen et al. 2017), with the exception of the position of *Haematopinus macronemis*. However, this placement is the only part of the tree that is not well supported, and reasons for incongruence are unclear, which could be, e.g. slightly different choice of phylogenetic markers. However, in general, the approach worked very well, and for 951 of 954 USCOs, nearly complete exonic data could be retrieved.

## Discussion

Patchwork is a new software for quickly mining phylogenetic markers from WGS data. Since Patchwork can retrieve homologous regions even in distantly related taxa, this program lends itself especially well for recovering phylogenetic markers for phylogenomic studies. It is simultaneously an efficient way for increasing marker occupancy in poorly assembled genomes and/or in the presence of multilocus exons. Finally, Patchwork allows the user to combine 2 different data types—i.e. transcriptomic and genomic data—into a single data set, thus further enabling an even larger taxon sampling and encouraging data reusability.

Special consideration should be taken to avoid the creation of chimeric sequences. One way in which such sequences may arise is when orthologous (i.e. genes related via a speciation event) and paralogous (i.e. genes related via a gene duplication event) sequences are merged together. To circumvent this issue, we recommend that the user limits the use of reference sequences to near-USCOs. Different lineage-specific sets of such USCOs are available based on carefully analyzed sets of homologous genes from a curated database (Manni et al. 2021). Besides their use in evaluating the quality of genomic and metagenomic data, USCOs became also prominent as preselected marker sets in phylogenomic analyses (Sahbou et al. 2022) and have been recently proposed as a unifying framework for DNA-based species delimitation (Dietz et al. 2023). Many programs, e.g. the aforementioned program BUSCO, exist for retrieving such sequences from an already assembled genome, and these could be used as reference sequences (Waterhouse et al. 2018). Additionally, several downstream analysis tools are available to control for the presence of possible cross-contamination, (unexpected) paralogous copies, or other artifacts confounding systematic studies (Lozano-Fernandez 2022). To control for the possible artifactual inclusion of stretches of noncoding sequences, the tool PREQUAL could be used to detect such and remove such regions (Whelan et al. 2018). Finally, multiple alignment tools such as MACSE (Ranwez et al. 2018) can be used to deal with putative confounding problems from the occurrence of premature stop codons, which might occur when working with data with coverage genomic data.

The accuracy and the robustness of the results depends on how closely related the target and the reference species under study are. The difficulty stems from the ability to accurately predict noncoding regions in aligned contigs; because alignment trimming relies on gap-excluded identity, choosing the correct cutoff threshold becomes increasingly easier as the level of identity approaches 100% (the identity of noncoding regions is likely to stay the same, while the identity to coding regions increases). On the upside, high-quality genomes for practically all major lineages exist and are readily available online (Formenti et al. 2022). Moreover, and not surprisingly, the coverage of the input read data sets correlates with the performance of retrieving single-copy marker genes. Similar to a previous study (Liu et al. 2021), we also find that a coverage of 10× and more should be targeted when designing genome skimming studies. However, as seen in the proof of principle, even lower coverages enable the construction of phylogenomic data matrices. For very low-coverage data sets, the read-based mode outperforms assembly-based analyses. For the latter, assembly size seems to be more important than contiguity.

In summary, Patchwork allows the retrieval of (putatively) single-copy genes from genome skimming data sets at different sequencing coverage with high computational speed. Availability and quality of biological specimens are becoming the major bottleneck for phylogenomic studies. Especially for phylogenomic studies relying on collection-based material, Patchwork offers a fast and efficient way for marker retrieval from short-read sequence data sets.

## Materials and Methods

Patchwork is implemented in Julia (Bezanson et al. 2017), a just-in-time (JIT)–compiled programming language that is typically faster than interpreted languages such as Python or R. Existing Julia bioinformatics packages such as BioAlignments.jl (https://github.com/BioJulia/BioAlignments.jl) and BioSequences.jl (https://github.com/BioJulia/BioSequences.jl) were used to speed up the development process. Patchwork is obtainable from GitHub (https://github.com/fethalen/Patchwork), is distributed under the GPLv3 license, and targets both Linux and macOS (Windows users may run Patchwork by using the Windows Subsystem for Linux).

In order to facilitate reproducibility, a Docker container (Merkel 2014) of Patchwork is also distributed via the BioContainers framework (da Veiga Leprevost et al. 2017). Similarly, we also provide an Apptainer definition file for users of the Apptainer/Singularity platform. Apptainer (formerly known as Singularity; Kurtzer et al. 2017) is another container platform that targets shared systems such as High-Performance Computing platforms, which are commonplace at universities today.

Most phylogenomic studies include more than a handful of taxa, and concatenating these manually gets increasingly tedious as the data set size increases. Therefore, Patchwork also includes a set of complementary tools for streamlining the downstream analysis. For example, the script multi_patchwork.sh lets the user run Patchwork on multiple input files and concatenate homologous sequences from different taxa into 1 file.

### Initial Alignment and Database Construction

First, all reference protein sequences, regardless of whether they are spread across multiple FASTA files or not, are pooled together into a single FASTA file, from which a DIAMOND database is created. There is also the option to use an existing DIAMOND-formatted database or a BLAST output file in a tabular format by using the --database or --tabular options, respectively. These files are both provided in the output of Patchwork and can thus be reutilized when trying out different parameters. In either case, DIAMOND's BLASTX algorithm is used to align translated nucleotide sequences to 1 or more reference protein sequences.

Like DIAMOND, Patchwork, by default, scores alignments using the substitution matrix BLOSUM62 (Henikoff and Henikoff 1996), a gap open penalty of 11, and a gap extension penalty of 1. Other built-in or custom substitution matrices may be used in place of the default option. User-chosen gap open penalties and gap extension penalties may also be set, as long as they fall within the limits set by the substitution matrix of choice. For the users’ convenience, Patchwork supports a number of different DIAMOND options that can usually be provided in the same manner as in DIAMOND itself.

For all Patchwork benchmarks, we observed that disabling DIAMOND's tantan masking (Frith 2011), by setting --masking 0, as described in Table 2, yielded higher query coverages. This effect was more pronounced for read data sets but could also be detected in assembled data sets. On the other hand, the number of exact matches in all aligned positions (i.e. percent identity) between the query and the reference decreased slightly. When combining both measures, however, disabling tantan masking improved the overall results.

Since the alignment search is likely to result in more than 1 hit per reference region, certain measures are taken to ensure that none of these hits are overlapping: They are, “hit stitching” (also known as contig or exon stitching; i.e. merging of overlapping regions), removal of unaligned residues, and concatenation of nonoverlapping regions.

### Hit Stitching

During “hit stitching,” all alignments made between the query region and the target sequence are merged in a way such that only the highest-scoring segment pair (HSP) for each region is retained. This results in a single, continuous sequence, and, as a consequence, some hits may be removed entirely (see also Fig. 1).

The “hit stitching” algorithm works as follows: First, query regions are sorted according to how they align to the target sequence—from first to last—and are added to the stack. Next, each pair of query regions on the stack is checked for overlaps. In case of an overlap, first, all regions are sorted by their first and last position at which they align to the reference sequence. The first region is added to the stack. Its start and end coordinates are then compared with those of the following region to check if they are overlapping. If they are not overlapping, the next adjacent region is added to the stack and compared with the following region. If they are overlapping, however, the region that is currently at the top of the stack is removed. The overlapping parts of this region and the next region are realigned to identify the best-scoring sequence at that particular interval. Then, based on the realignment score, the sequences are sliced such that the best-scoring sequence is retained at the overlapping region and so that the nonoverlapping, flanking parts of both regions, if existing, are preserved as well. Thus, a maximum of 3 sliced region parts are then added to the stack as new, separate regions: The sequence part preceding the overlap, which originates from the first region, the highest-scoring sequence at the overlap, which may be from either of the 2, and the sequence part that follows the overlap, which originates from the second region. The algorithm then continues in the same manner, comparing the topmost region of the stack with the following region, until all overlaps are removed and all regions have been added to the stack. This procedure may require multiple iterations, since in every run, only each pair of consecutive regions are compared and merged.

Different aligned regions from the same contig are allowed to be stitched together. While “hit stitching” may result in the creation of chimeric sequences (i.e. 2 or more biological sequences incorrectly joined together), this procedure has the potential to increase coverage and to (correctly) join 2 or more regions that are located on separate contigs due to incomplete assembly or sequencing errors.

### Alignment Masking

At this step, unaligned residues, ambiguous amino acid characters, and stop codons (also known as “termination codons”) are all removed from the resulting query sequence. Query sequences may contain residues that do not align to any particular region of the subject sequence. Such regions may be noncoding regions or simply insertions. In either case, unaligned residues are removed on the basis that inserts are less likely to constitute phylogenetically informative sites and risks introducing untranslated regions and therefore biasing the downstream analysis. Similarly, ambiguous amino acids are most likely noninformative, and stop codons are a clear indicator that noncoding characters have been included in the alignment. Although such regions are likely to be removed in the subsequent step (see above), the user may choose to keep stop codons and/or ambiguous amino acid characters by providing the flags --retain-stops and/or --retain-ambiguous.

### Sliding Window–Based Alignment Trimming

One side effect of aligning translated nucleotide sequences to amino acid sequences is that one might recover noncoding portions of DNA, provided that the following 2 conditions are fulfilled: (i) the noncoding DNA is located in between 2 or more coding portions and (ii) there is a sequence region in the reference sequence that the noncoding region can align to. In the resulting alignment, noncoding portions are characterized by many indels, intercepted by occasional matches. The alignment of noncoding portions of DNA can already be observed in the alignments produced by DIAMOND, and thus, this side effect does not stem from Patchwork itself. In fact, the Patchwork algorithm will only include noncoding parts if nothing else aligns better to the affected region of the reference sequence.

To mitigate this effect, we have implemented a sliding window–based alignment trimming approach to rid the alignments from these unwanted regions. This works by scanning the alignment from left to right, cutting all regions where the average distance between query and reference is above the user-provided distance threshold. The window size and the distance threshold can both be set by the user, but need not be, since we implemented default values for both. This step can also be skipped over in its entirety. This approach tries to avoid cases where a single bad, but correct, match would have otherwise been cut out.

### Concatenation and Realignment of Remaining Regions

Finally, the resulting set of ordered, nonoverlapping sequence regions are concatenated into 1 continuous sequence. The concatenated sequence is then realigned to the reference to obtain the final output sequence and alignment score.

### Benchmark

Patchwork v.0.5.1 was continuously run using Julia v.1.8.2 and DIAMOND v.2.0.13 (Buchfink et al. 2021), with the options --ultra-sensitive --frameshift 15 --masking 0. All analyses were performed on the high-performance computing cluster maintained by the *Gesellschaft für wissenschaftliche Datenverarbeitung mbH Göttingen* (GWDG), running the Scientific Linux release 7.9 (Nitrogen) operating system with a Linux kernel of version 3.10.0. All runs were allocated 32 Intel Xeon Platinum 9242 CPUs running at 2.30 GHz. Elapsed time was calculated as reported by Slurm.

### Effect of Genome Fragmentation on Accuracy

A publicly available set of Illumina short-read sequences of *D. gyrociliatus* (Martín-Durán et al. 2021) was used for the query set. We used SPAdes v.3.15.3 to generate the de novo assembly, using a *K*-mer size of 55. A set of 815 *D. gyrociliatus* USCOs retrieved from the published high-quality genome assembly (GenBank, accession: GCA_904063045.1) served as the reference.

### Effect of Reference Divergence

We reused the de novo assembly from the previous evaluation for the query, while a set of 957 near-USCOs from the annotated genome of the leech *H. robusta* (GenBank, accession: GCA_000326865.1) were used as reference sequences. We used the same parameter settings described above. For this evaluation, we did not use Patchwork's own accuracy and completeness assessment, because the true number of identical matches and the amount of query coverage are not known between the 2 divergent species *D. gyrociliatus* and *H. robusta*. We therefore chose to compare the recovered markers to a subset of the *D. gyrociliatus* USCOs described in the previous benchmark. More specifically, only those *D. gyrociliatus* USCOs that produced a hit when searching against the *H. robusta* USCOs with DIAMOND v2.0.13 in ultrasensitive mode were used, since only these were considered “recoverable” in this setup. The resulting *D. gyrociliatus* USCO set contains 778 sequences; 37 sequences were discarded. The set of recovered markers was searched against the reference USCO set using DIAMOND in --ultra-sensitive mode. For each reference sequence, we retrieved only the marker that produced the highest bit score during the alignment step. We then evaluated percent identical positions out of all aligned positions as well as percent of reference sequence positions covered by the recovered markers.

### Program Comparison

In order to generate data sets at different sequencing coverages, we subsampled the trimmed *D. gyrociliatus* reads downloaded from NCBI GenBank. Corresponding read pairs were selected randomly from the paired-end data. Subsampling was done using Subsample.jl, a Julia package distributed together with Patchwork. The resulting data sets have coverages of 1×, 3×, 5×, 10×, 20×, and 40×.

For each of the data sets, we produced a short-read-only de novo assembly, as ALiBaSeq is designed for assembly data, while aTRAM 2 requires read data and Patchwork can process both. We used the assembler SPAdes v.3.15.3 (Nurk et al. 2013), with a *K*-mer size of 33, and the quality of the assembly was assessed using QUAST v.5.0.2 (Gurevich et al. 2013). We aligned the *D. gyrociliatus* reads and assemblies against the same set of *H. robusta* USCOs mentioned before.

ALiBaSeq v.1.2 was run with the *D. gyrociliatus* assemblies described above. The program requires BLAST; the version here used was 2.11.0. The program builds a database from the *D. gyrociliatus* sequences and searches this database with the *H. robusta* sequences before stitching the hits together. We set the parameters according to the guide for a protein-based search without reciprocal search, as explained in their documentation on GitHub (see the README file): -x a [extract all hits and join into (super)contigs] -f S [single alignment table (TBLASTN result file)] -e 1e-10 [*e*-value cutoff for further processing of TBLASTN hits] -c 1 [extract single best (super)contig] --amalgamate-hits [scoring scheme for (super)contigs] --is [enable contig stitching] –ac aa-tdna [search protein “baits” (*H. robusta* USCOs) against tDNA “target” database (*D. gyrociliatus* reads)].

We ran aTRAM v.2.4.3 with the sampled *D. gyrociliatus* read data sets. The program further requires BLAST, as well as a de novo assembler, and exonerate. We used BLAST v.2.11.0 and exonerate v.2.2.0 (Slater and Birney 2005) and employed SPAdes v.3.15.3 for the assembly step. The full aTRAM 2 pipeline consists of 3 consecutive steps: Firstly, the preparation of a database from the *D. gyrociliatus* reads, secondly, the assembly of different loci, and lastly, a reference-guided stitching process. The parameter settings for the core module of aTRAM 2 as well as the stitcher were as follows: --evalue 1e-10 --file-filter “*.filtered contigs.fasta” --overlap N.

Patchwork v.0.5.1 was run with both the sampled *D. gyrociliatus* read data sets and the assemblies we produced for these sampled read data. We ran the uncompiled program using Julia v.1.8.2 as well as the compiled version on each data set in order to perform runtime comparisons. Patchwork achieves all its objectives in a 1-step procedure, i.e. can be called with a single command, unlike ALiBaSeq and aTRAM 2. The program builds a DIAMOND database from the *H. robusta* sequences and, after obtaining *D. gyrociliatus* hits, proceeds to stitch them together. All nondefault parameter settings for Patchwork were as described above. They were used for both read-based runs and assembly-based runs.

We ran the 3 programs with their respective parameter settings on the different *D. gyrociliatus* data sets against the *H. robusta* USCO set described above, which contains 957 sequences. The aTRAM 2 run for the 40× coverage read data set was ended prematurely because it had not terminated after 5 d. In a following step, the recovered markers produced by each program for each data set were evaluated with respect to completeness and accuracy of the resulting sequences by comparing them to the same set of 778 *D. gyrociliatus* USCOs mentioned above, again because only this subset could be recovered by the programs in this setup. ALiBaSeq and aTRAM 2 output DNA sequences that contain the ambiguous nucleotide N in all positions that could not be recovered during stitching. These N were removed for the subsequent evaluation steps because they distort the query coverage measure; the amount of a reference sequence covered by the recovered marker is artificially increased due to the uninformative inserted N.

Completeness and accuracy were measured jointly as percent identical aligned positions multiplied with the total amount of aligned, or recovered, positions (here called *p*_identical, cov_):


$$p_{\mathrm{identical},\mathrm{cov}}=\frac{n_{\mathrm{match}}}{n_{\mathrm{aligned}}}\cdot \mathrm{cov}.$$

$$\mathrm{cov}=\frac{\sum s_{\mathrm{recovered}}(\mathrm{length}(s_{\mathrm{recovered}}))}{\sum s_{\mathrm{USCOs}}(\mathrm{length}(s_{\mathrm{USCOs}}))}.$$

*n*_match_ being the total number of exact matches in all alignments between recovered markers and reference USCOs and *n*_aligned_ the total number of aligned, i.e. nongap, positions. The coverage cov was computed as the ratio of the total lengths of all recovered markers *s*_recovered_ and all reference USCOs *s*_USCOs_. We chose to combine the measures for accuracy of the recovered markers, i.e. percent identical out of all aligned positions, and completeness or query coverage, i.e. percent recovered positions, in order to avoid a distorted outcome. For example, a program might recover only a very small number of markers but these with high percent identity, such that using only the percent identity measure would have resulted in an overestimation of the program's performance.

### Patchwork in a Phylogenomic Context

We retrieved the raw reads from the NCBI Sequence Read Archive (SRA accession SRR5088465, SRR5088468, SRR5308129 SRR5308123, SRR5088469 SRR5088471, SRR5088472, SRR5088473, SRR1182279, SRR5308136, SRR5308138, SRR5088474, SRR5088475, SRR5308112, and SRR5088466) using prefetch, vdb-validate, and fasterq-dump (with the flag --split-spot), all from the NCBI SRA toolkit (Leinonen et al. 2011). We ran Patchwork v.0.5.1 with each of the specimens as query input and a set of 957 near-USCOs from the leech *H. robusta* as reference sequences (see Table 2 for parameter settings). A multiple sequence alignment (MSA) was constructed for each of these 957 loci using MAFFT (Katoh and Standley 2013) with the options –globalpair --ep 0.123. The resulting alignments were trimmed with trimAl (Capella-Gutiérrez et al. 2009), removing all positions with more than 90% gaps but retaining at least 60% of each alignment (options -gt 0.9 and -cons 60, respectively). We used FASconcat-G (Kück and Longo 2014) to concatenate the trimmed alignments into a supermatrix. This supermatrix was then input into IQ-TREE 2 (Minh et al. 2020), alongside its corresponding gene partition file, to reconstruct the phylogeny using the maximum likelihood (ML) approach. We ran IQ-TREE 2 with extended model selection and tree inference, calculating 1,000 replicates for the ultrafast bootstrap (command line options -m MFP and -B 1000, respectively).

## Data Availability

The data underlying this article are available via GitHub at https://github.com/animal-evolution-and-biodiversity/benchmarking-patchwork. Patchwork is distributed under the GPLv3 license via GitHub at https://github.com/fethalen/patchwork.
